# Effects of Different Types of Water and Nitrogen Fertilizer Management on Greenhouse Gas Emissions, Yield, and Water Consumption of Paddy Fields in Cold Region of China

**DOI:** 10.3390/ijerph16091639

**Published:** 2019-05-10

**Authors:** Tangzhe Nie, Peng Chen, Zhongxue Zhang, Zhijuan Qi, Yanyu Lin, Dan Xu

**Affiliations:** 1 School of Water Conservancy and Civil Engineering, Northeast Agricultural University, Harbin 150030, China; nietangzhe@neau.edu.cn (T.N.); liuwanning@neau.edu.cn (P.C.); xd_neau@126.com (D.X.); 2 Key Laboratory of Agricultural Water Resource Use, Ministry of Agriculture, Harbin 150030, China; 3 College of Engineering, Heilongjiang Bayi Agricultural University, Daqing 163319, China; Linyanyu@163.com

**Keywords:** paddy fields, irrigation mode, greenhouse gas, yield, water productivity, comprehensive assessment

## Abstract

Water management and nitrogen (N) fertilizers are the two main driving factors of greenhouse gas emissions. In this paper, two irrigation modes, controlled irrigation (CI) and flood irrigation (FI), and four nitrogen fertilizer levels (N0: 0, N1: 85, N2: 110, and N3: 135 kg·hm^−2^) were set to study the effect of different irrigation modes and N fertilizer amount on greenhouse-gas emissions of paddy fields in cold region by using the static chamber-gas chromatograph method; yield and water consumption were also analyzed. The results showed that, compared with FI, CI significantly reduced CH_4_ emissions by 19.42~46.94%, but increased N_2_O emissions by 5.66~11.85%. Under the two irrigation modes, N fertilizers could significantly increase N_2_O emissions, but the CH_4_ emissions of each N treatment showed few differences. Compared with FI, appropriate N application under CI could significantly increase grain number per spike, seed-setting rate, and 1000-grain weight, thus increasing yield. Under the two irrigation modes, water consumption increased with the increase of N application rate, and the total water consumption of CI was significantly lower than that of FI. The global warming potential (GWP) of CI was significantly smaller than that of FI. The trend of GWP in each treatment was similar to that of CH_4_. Through comprehensive comparison and analysis of water productivity (WP), gas emission intensity (GHGI), and the yield of each treatment, we found that CI+N2 treatment had the highest WP (2.05 kg·m^−3^) and lowest GHGI (0.37 kg CO_2_-eq·kg^−1^), while maintaining high yield (10,224.4 kg·hm^−2^). The results of this study provide an important basis for guiding high yield, water-savings, and emission reduction of paddy fields in cold regions.

## 1. Introduction

Methane (CH_4_) and nitrous oxide (N_2_O), as the major greenhouse gases emitted from farmland soils, have an important impact on global climate change. The fifth assessment report of the Intergovernmental Panel on Climate Change (IPCC) pointed out that the global warming potential (GWP) of CH_4_ and N_2_O was 21 times and 310 times that of CO_2_, respectively, on the 100-year scale [1]. In 2010, agricultural non-CO_2_ greenhouse gas emissions were about 5.2–5.8 Gt CO_2_-eq. Rice fields were the main source of agricultural CH_4_, and N_2_O was also emitted from paddy fields. Even though N_2_O emissions were much lower than CH_4_, N_2_O emissions are likely to increase with the increasing N application rate and the anaerobic–aerobic cycle of paddy fields [2,3]. Non-CO_2_ greenhouse-gas emissions of rice fields were about 493–723 Mt CO_2_-eq, accounting for 9–11% of total agricultural emissions [4]. Previous studies showed that greenhouse gas emissions from rice fields were mainly affected by climatic conditions, soil properties, water management, and tillage measures, and these factors have great potential to reduce greenhouse gas emissions [5,6,7].

In rice production, water management and nitrogen (N) fertilizers are the two main driving factors of greenhouse gas emissions [8,9,10]. N application can increase emissions of N_2_O in paddy fields, but the mechanism of N fertilizers on CH_4_ emissions is more complicated. There is still much controversy about whether N promotes or inhibits CH_4_ emissions [11,12]. In terms of water management, controlled irrigation (CI) can reduce CH_4_ emissions compared with flood irrigation (FI); however, at the same time it increases N_2_O emissions [13,14]. Heilongjiang Province, as the main rice-producing area in cold region, has increased its rice-planting area by about 3.4 times in the past 20 years [15]. The increase in its rice-planting area was larger than in other provinces in China, with greatly increased agricultural water consumption and aggravated water shortages. Popularizing the CI rice mode can reduce water consumption per unit area compared with the traditional FI and effectively alleviate this contradiction [16]. Therefore, it is of great significance to study the optimal water-saving and emission-reduction irrigation mode in paddy fields under different water and N management types for food security, reducing greenhouse emissions and alleviating the shortage of water resources in China.

At present, there are many scientific studies on greenhouse gases in paddy fields in China, but they are mainly concentrated in southern areas [17,18,19]. The cold rice-planting area is located at a high latitude with insufficient water and heat resources [20,21]. Temperature increases slowly during rice’s early growth stage, high-temperature periods have a short duration during the middle-growth stage, and temperature decreases fast during the late stage. The planting area of rice in cold area increases year by year [15]. These factors have increased the uncertainty relationship between rice yield, water-saving, and greenhouse gas emissions. In this paper, the effects of different N application rates and water management on CH_4_ and N_2_O emissions, yield, and water consumption of paddy fields in cold regions were investigated by field-plot experiments, and the GWP, gas emission intensity (GHGI), and water productivity (WP) were comprehensively evaluated. The aim of this study is to evaluate the ecological environmental effect of paddy fields and the effect of water-savings, study increased yield under different water and N management types, and provide the basis for water-savings, yield-increases, and emission reductions in the paddy fields of cold regions.

## 2. Materials and Methods

### 2.1. Experimental Design

**Figure 1:**
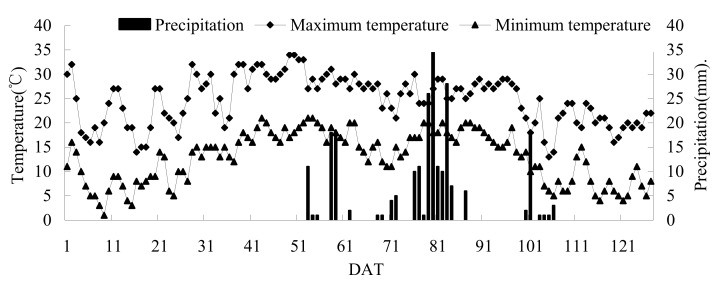
Changes of air temperature and rainfall during the growth period of rice. DAT: days after transplanting, same as below.

The experiment was conducted at the National Rice Irrigation Experiment Center (127°40′ E, 46°57′ N) in Heilongjiang Province in 2017, and rice was planted in the experimental area for more than 20 years. Average annual temperature is 2~3 °C, while accumulated temperature is 2300~2500 °C. Average annual rainfall is 500~600 mm. The frost-free period is 128 days. The experimental area has a temperate continental monsoon climate. The soil is albic rice soil, the soil-bulk density is 1.01 g/cm^3^, and soil porosity is 61.8%. Basic physicochemical properties of soil are as follows: organic matter content, 41.8 g/kg; pH, 6.4; total N, 15.06 g/kg; total P, 15.23 g/kg; total K, 20.11 g/kg; alkali hydrolysable N, 198.29 mg/kg; available P, 36.22 mg/kg; and available K, 112.06 mg/kg. The meteorological data of the rice-growth period were recorded by automatic weather station DZZ2X (Tianjin Meteorological Instrument Factory), and are shown in Figure 1.

CI and FI were used in the experiment, and the corresponding water management is shown in Table 1. CI had no water layer for the rest of the growth period except for the regreening period and each fertilizer application. The surface-water layer of the field was irrigated to a depth of 1 cm before each fertilizer application. Four kinds of N application levels were selected: no N treatment (N0: 0 kg·hm^−2^), low N application level (N1: 85 kg·hm^−2^), normal N application level (N2: 105 kg·hm^−2^), and high N level (N3: 135 kg·hm^−2^). A total of eight treatments were performed, and each treatment was repeated three times. The length and width of each plot were 10 m, respectively, and the area was 100 m^2^. Each plot had a water meter, and the irrigation and drainage of each plot were separated. The cement ridge and plastic clapboard were used for seepage between the plots to prevent moisture and fertilizer exchange.

The fertilizers tested were urea (N, 46%), superphosphate (P_2_O_5_, 12%), and potassium chloride (K_2_O, 60%). For the N fertilizer, the ratio of basal fertilizer:tillering fertilizer:spikelet-promoting fertilizer:spikelet-preserving fertilizer was 4.5:2:1.5:2. P_2_O_5_ 45 kg·hm^−2^ and K_2_O 80 kg·hm^−2^ were used for each treatment, K_2_O fertilizer was applied twice as basal fertilizer and 8.5 leaf age, respectively; the ratio before and after was 1:1. P_2_O_5_ fertilizer was applied to the basal fertilizer once. The tested rice was Longqing 3. According to the technical requirement of rice seedlings in the cold region, sowing began when air temperature was stable at 5~6 °C, and the seedbed-soil temperature was above 12 °C. Pregerminated seeds were cultivated into the seedlings in a soil-filled seedbed. Sowing started on 20 April and seedlings were transplanted on 17 May. Planting density was 30 × 10 cm. Technical conditions such as seedling raising, transplanting, density, fertilization, and pesticide use were the same for each plot. Rice was harvested on 20 September.

### 2.2. Gas Sampling and Analysis

Field sampling of CH_4_ and N_2_O was carried out from the beginning of seedling transplanting to harvest for about once a week. On cloudy or rainy days, sampling was postponed until the next sunny day. The number of sampling times was increased during the rapid rice-growth period. There were a total of 17 sampling events in this experiment. The static-chamber method was used for sampling. The length and width of the chamber and the stainless-steel base were 25 × 25 cm. The chamber was made of 5-mm-thick plexiglass, which was covered with an insulation board and tin foil to prevent solar radiation from causing temperature changes inside the chamber. A chamber with 60-cm height was used in the early growth stage of rice, and a chamber with 110-cm height was used in the late growth stage. A 12-V fan was installed inside each chamber to ensure the gas inside was evenly mixed. An electronic thermometer probe (0.1 °C) was equipped inside the chamber to correct emission errors caused by the rise of temperature. The sampling port was set on the side of the chamber, and the sampling tube was inserted into the chamber for 20 cm. The gas was sampled by a 50-mL syringe, and was then injected into the gas-collection bag (E-Switch). The upper part of the base had a 5-cm-wide and 5-cm-high sink, and the sink was filled with water before gas sampling to ensure that there was no gas exchange between the chamber and the external environment.

Samplings time were from at 10:00 to 12:00 h, with sampling once at 0, 10, 20, and 30 min, respectively, during the time of chamber closure. Before each sampling, 30 mL gas was first extracted from the sampling tube with a syringe, and then injected back into the chamber to reduce the error caused by the nonuniform mixture of gas in the sampling tube. After sampling, the air bag was taken directly to the laboratory for analysis. Gas concentration was manually analyzed by gas chromatograph (SHIMADZU GC-2010plus, Japan).

The emission flux of CH_4_ and N_2_O was calculated with the following formula [7]:

(1) $$F=\rho h\cdot \frac{dC}{dt}\cdot \frac{273}{273+t}$$

where *F* is the CH_4_ flux (mg·m^−2^·h^−1^) or N_2_O flux (ug·m^−2^·h^−1^); $\frac{dC}{dt}$ is the slope of curve of gas concentration versus time; *h* is the effective height of the chamber (m); ρ is gas density at the standard state (kg·m^−3^); and *T* is the average temperature inside the chamber (°C).

GWP and GHGI were used to assess the greenhouse-gas effects. Taking 100a as the time scale, the GWP of CH_4_ and N_2_O gas per unit mass was 21 and 310 times that of CO_2_, respectively (IPCC, 2014); its unit is kg CO_2_-eq·hm^−2^. GHGI represents the GWP per unit rice yield; its unit is kg CO_2_-eq·kg^−1^.

### 2.3. Yield and Components

In the maturity period, a 1-m^2^ block of well-grown and evenly grown rice plants from each plot was collected for yield measurement. Rice yield per unit area was calculated according to 14.5% moisture content. Spikes per unit area, grain number per spike, seed-setting rate, and 1000-grain weight were also counted at the same time.

### 2.4. Water Consumption and Water Productivity

Soil-moisture content was measured with a TPME-PICO64/32 soil-moisture analyzer when there was no water layer in the field. When there was a water layer, the water depth of three points in each plot was recorded at 08:00 h every day, and the average value was then calculated. When the height of the water layer was greater than the upper limit of water, drainage would be carried out and the depths of the water before and after drainage were recorded. Water consumption was calculated by the water-balance equation:

(2) $$ET=P+I+K+W_{1}-R-D-W_{2}$$

where *ET* is water consumption (mm); *P* is precipitation (mm); *I* is the irrigation water amount (mm); *K* is groundwater recharge (mm); *W1* and *W2* are soil water storage before and after the growth period (mm), respectively; R is the drainage amount (mm); and *D* is the percolation amount (mm).

Because groundwater in the experiment area is relatively deep, groundwater recharge does not exist, so *K* = 0. The soil percolation amount in the paddy fields was taken as an average value of experiment area based on the thesis of Guo [22].

The *WP* was obtained from rice-grain yield (*Y*) per unit of water consumption. The calculation formula was as follows:

(3) WP=Y/ET

where *WP* is water productivity (kg·m^−3^).

### 2.5. Data Analysis

To test the differences between treatments, the data were analyzed using analysis of variance with SPSS 17.0 (IBM Corp., New York, NY, USA). The treatment means were compared with a least-significant-difference (LSD) test.

## 3. Results and Analysis

### 3.1. CH_4_ Emissions

**Figure 2:**
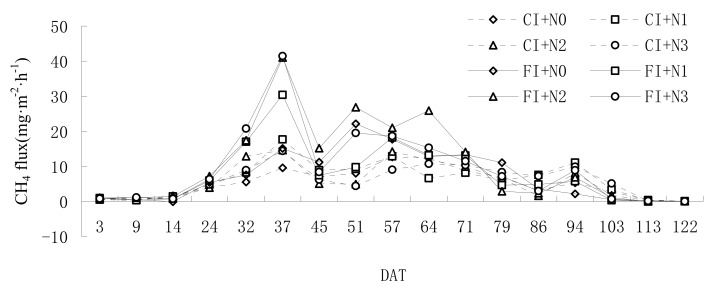
Change of CH_4_ emission flux in each treatment.

As shown in Figure 2, there were three emission peaks during the growth period: in the later tillering drainage, jointing and booting stage, and milk maturity stage. The average flux of each peak decreased as days after transplanting (DAT) increased. Under the same N application condition, the CH_4_ emission flux of each treatment under CI was lower than that under FI except for the late growth stage of rice, which showed that CI had an overall inhibition effect on the CH_4_ emission flux. The peak value of FI+N3 emission flux was 41.45 mg·m^−2^·h^−1^, which was 135.24% higher than the peak of CI. The second peaks of FI and CI occurred at 51 and 57 DAT, respectively. This may be due to better soil aeration at the tillering stage under CI inhibiting the amount of methanogen [19], and drainage practice in later tillering further decreasing the amount of methanogen. After rewatering at the beginning of the jointing and booting stage, the amount of methanogen was difficult to rapidly increase in a short time. This resulted in the second peak of CI treatments later than FI for six days, and the emission peak value was less than FI. The third emission peak of CH_4_ emission was at 94 DAT. In this peak, the CH_4_ flux of CI was slightly higher than that of FI. This may be because in the late growth of rice, the root system of control irrigation was more developed, root exudates provided more substrates for the production of CH_4_, and the developed roots were beneficial for the release of CH_4_ through the plant pathway.

**Figure 3:**
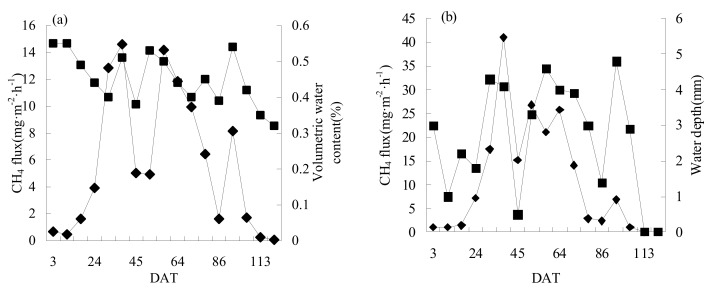
Relationships between (**a**) CH_4_ flux and volumetric water content in CI+N2, and (**b**) CH_4_ flux and water depth in FI+N2.

CI+N2 and FI+N2 were selected to analyze the relationships between CH_4_ flux and field-soil water conditions (Figure 3). There was a significant positive correlation between CH_4_ flux and soil water content (*p* < 0.05) in CI+N2 except in the regreening stage, while there was a significant positive correlation between methane-emission flux and water depth during the whole growth period of rice under FI (*p* < 0.05). Under both irrigation modes, the increase of water content and water depth promoted the increase of CH_4_ emission flux.

By comparing the CH_4_ emissions of each treatment in Table 2, under the same N application, CH_4_ emissions of CI+N3, CI+N2, CI+N1, and CI+N0 decreased by 37.98%, 46.94%, 19.42%, and 28.23%, compared with FI+N3, FI+N2, FI+N1, and FI+N0, respectively, indicating that CI under the same N level could significantly reduce CH_4_ emissions of paddy fields. For CH_4_ emissions of CI treatments, CI+N3, CI+N2, and CI+N1 were significantly higher than those of CI+N0, but differences between CI+N3, CI+N2, and CI+N1 were not significant. CH_4_ emissions of CI+N1 were the largest (189.79 kg·hm^−2^), which were 4.95%, 8.56%, and 20.30% higher than CI+N3, CI+N2, and CI+N0, respectively. For CH_4_ emissions of FI treatments, CH_4_ emissions first increased and then decreased with the increase of N application. CH_4_ emissions of FI+N2 were the largest (329.48 kg·hm^−2^), which were 13.01%, 39.88%, and 49.89% higher than those of FI+N3, FI+N1, and FI+N0, respectively. CH_4_ emissions of FI+N3 and FI+N2 were significantly higher than those of FI+N1 and FI+N0. However, the difference between FI+N3 and FI+N2 was not significant. Results showed that N application promoted CH_4_ emissions under FI, and there existed a CH_4_ emission threshold with the increase of N application. In summary, under two irrigation modes, N application promoted CH_4_ emissions in paddy fields, and CI could significantly reduce CH_4_ emissions.

### 3.2. N_2_O Emissions

**Figure 4:**
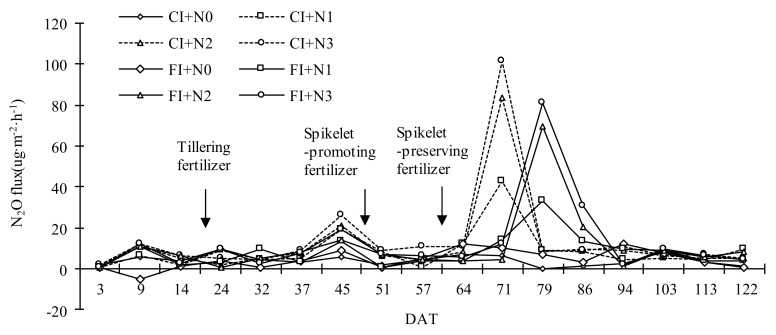
Change of N_2_O emission flux in each treatment.

From Figure 4, we can see there were two emission peaks during the rice-growth period, which were in the later tillering drainage and the heading–flowering stage, respectively. In other periods, the N_2_O emission flux fluctuated at a smaller value. At each N_2_O flux peak, the N_2_O flux of CI was generally higher than that of FI. The first smaller N_2_O emission peak occurred on 45 DAT, which was possibly because of the reduction of soil-moisture content during late tillering drainage, which increased the available oxygen in the soil and finally promoted the emission of N_2_O. In addition to CIN0 and FIN0, the second emission peaks were found at 71 and 79 DAT, respectively. Fluctuation ranges were 6.63~101.68 ug·m^−2^·h^−1^ and 10.11~81.07 ug·m^−2^·h^−1^, respectively. FI peak lagged behind that of CI; this may because the thicker water layer of FI was not conducive to N_2_O production and it blocked the diffusion of N_2_O from the soil to the atmosphere. The peak of N_2_O emissions did not appear after the application of tillering fertilizer and spikelet-promoting fertilizer, but there was an emission peak after applying spikelet-preserving fertilizer. This may be due to the low soil temperature at the tillering stage, and the low nitrification and denitrification reaction in the soil leading to a smaller N_2_O flux. Continuous cloudy and rainy days occurred after spikelet-promoting fertilizer application (Figure 1). As a result, the effect of N application on N_2_O emissions was not captured. After applying the spikelet-developing fertilizer, the weather was sunny, effective heat radiation received by the field was larger, and suitable soil and climate conditions promoted N_2_O emissions.

**Figure 5:**
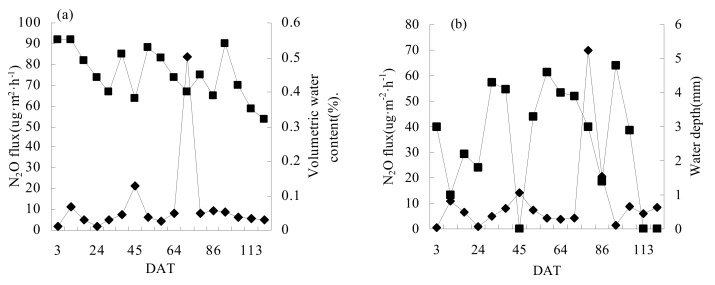
Relationships between (**a**) N_2_O flux and volumetric water content in CI+N2, and (**b**) N_2_O flux and water depth in FI+N2.

The relationships between N_2_O flux and field-soil water conditions are shown in Figure 5. There was a trade-off relationship between N_2_O and soil-water content in CI+N2 treatment except in the regreening stage and yellow-maturing stage, but correlation analysis showed that the relationship between them was not significant (*p* = 0.095). Except for 79 DAT N fertilizer application, there was a significant negative correlation between N_2_O and water depth (*p* < 0.05) in FIN2. In both irrigation modes, reducing soil-moisture content and field-water depth promoted N_2_O emission. However, the promotion effect was only significant under FI.

As shown in Table 2, under the same N application, N_2_O emissions of CI+N3, CI+N2, CI+N1, and CI+N0 increased by 11.85%, 10.55%, 9.88%, and 5.66% more than those of FI+N3, FI+N2, FI+N1, and FI+N0, respectively, indicating that CI treatments could significantly increase N_2_O emissions. Among CI treatments, N_2_O emissions of CIN3 were the largest (0.44 kg·hm^−2^), at 22.91%, 62.62%, and 207.73% higher than those of CI+N2, CI+N1, and CI+N0, respectively. Among FI treatments, the N_2_O emissions of FI+N3 were the largest (0.39 kg·hm^−2^), at 21.48%, 59.75%, and 190.69% higher than those of FI+N2, FI+N1, and FI+N0, respectively. There were significant differences between the levels of N application among both CI and FI treatments, N_2_O emissions increased with the increase of N application, and the increase of N_2_O emissions under CI was larger than that under FI. In summary, under the two irrigation modes, increasing the amount of N application would significantly promote N_2_O emissions. Compared with FI, CI significantly increases N_2_O emissions.

### 3.3. Yield and Its Components

As shown in Table 3, under the same N application, the yield of CI treatments was 5.53~27.01% higher than that of FI treatments. Among them, the increase of CI+N2 is the largest, which indicates that CI has a good effect on increasing yield. Under FI, yield increased with the increase of N application, and maximum yield was 8049.78 kg·hm^−2^ when N application was 135 kg·hm^−2^. Under CI, yield initially increased and then decreased with the increase of N application. When N application was 110 kg·hm^−2^, maximum yield was 10,224.4 kg·hm^−2^. Therefore, from the perspective of irrigation mode and N application, the appropriate N application rate under CI was more conducive to a higher yield.

The yield components of two irrigation modes under different N applications are also analyzed in Table 3. For CI treatments, except for the seed-setting rate, the other three yield components had a threshold value with the increase of N application, which showed a trend to first increase and then decrease, resulting in the same trend of yield change. For FI treatments, the spikes per unit area and 1000-grain weight increased with the increase of N application, while the grain number per spike and seed-setting rate showed a tendency to rapidly increase and then slightly decrease, resulting in a continuous yield increase with the increase of N application. Under the same amount of N application, compared with FI treatments, the spikes per unit area of CI treatments decreased by 5.80~13.98%, while grain number per spike, seed-setting rate, and 1000-grain weight increased by 1.06~14.09%, 1.57~5.34%, and 1.52~2.49%, respectively. Compared with FI treatments, the increase of grain number per spike, seed-setting rate, and 1000-grain weight of CI treatments made up for the loss of panicle number per unit area, and eventually led to an increase in yield of CI treatments.

### 3.4. Water Consumption

**Figure 6:**
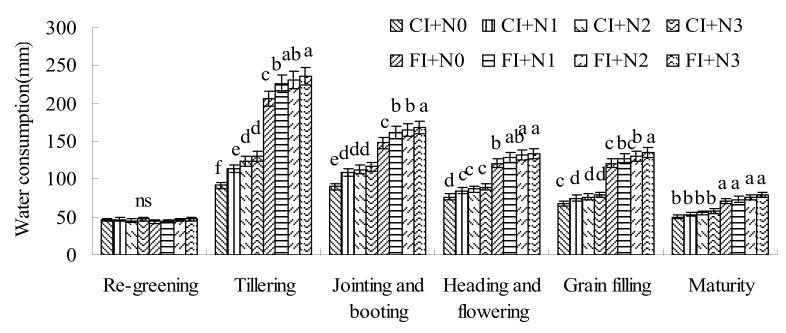
Water consumption during each growth period of rice. Note: Lowercase letters represent the differences between each treatment (*p* < 0.05), “ns” means being insignificant between each treatment.

As shown in Figure 6, the water-consumption trends of rice at different growth stages under different treatments were the same. Under two irrigation modes, the descending order of water consumption in different growth periods was: tillering stage > jointing and booting stage > heading and flowering stage > grain-filling stage > maturity stage > regreening stage. Due to the same water-management method in the regreening stage, and seedlings being smaller and the transpiration of plants being weaker, the water consumption of each treatment had no significant difference. In addition to the regreening stage, the water consumption of CI treatments during each growth stage of rice was significantly lower than that of FI treatments. Comparing the water consumption at different growth stages under the same N application rate, we can see that the difference of water consumption between CI and FI treatments was the largest during tillering stage. The water consumption of CI+N0, CI+N1, CI+N2, and CI+N3 decreased by 55.52%, 49.74%, 46.27%, and 45.08%, respectively, compared with FI+N0, FI+N1, FI+N2, and FI+N3. The water-saving effect of CI was the most obvious in this period. At the grain-filling, jointing and booting, heading and flowering, mature, and tillering stages, the differences between water consumption of CI and FI treatments decreased, and the water-saving effect of CI gradually became smaller. By comparing the relationship between water consumption and N application at different stages, it was found that the water consumption of each growth period increased with the increase of N application under both CI and FI. For CI, water consumption increased by 23.97~41.18%, 21.13~29.59%, 10.44~16.97%, 10.31~16.35%, and 6.19~15.77% in the tillering stage, the jointing and booting stage, the heading and flowering stage, the grain-filling stage, and the mature period under different N application rates compared with the CI+N0 treatment. However, for FI, water consumption increased by 9.69~14.34%, 9.49~13.62%, 6.63~10.2%, 4.97~11.85%, and 3.83~12.06%, respectively, compared with FI+N0 in the corresponding growth stages. By comparing the effects of N application on water consumption in different growth stages of rice under two irrigation modes, it could be seen that the effect of N application on water consumption under CI was more significant than that under FI.

From Table 2, under the same N application, the total water consumption of CI+N0, CI+N1, CI+N2, and CI+N3 decreased by 40.47%, 36.68%, 35.91%, and 34.89%, respectively, compared with FI+N0, FI+N1, FI+N2, and FI+N3; CI total water consumption was significantly lower than that of FI. Compared with FI, a water layer was not established in the paddy fields in each growth stage except the regreening stage for CI. CI reduced the evaporation of field water while meeting the rice-growth requirement, and achieved the goal of saving water. Among CI treatments, the maximum water-consumption treatment was CIN3 (5197 kg·hm^−2^), which was 3.98%, 7.84%, and 23.04% higher than that of CI+N2, CI+N1, and CI+N0, respectively. Among FI treatments, the maximum water-consumption treatment was FI+N3 (7982 kg·hm^−2^), which was 2.35%, 4.89%, and 12.50% higher than that of FI+N2, FI+N1, and FI+N0, respectively. Under the two irrigation modes, increasing the amount of N application would lead to an increase of water consumption. In summary, increasing N application significantly increased water consumption, but the water-consumption amount in each growth period of rice with different N applications could still be significantly reduced under CI with different N applications compared with FI; thus, total water consumption was reduced, and the effect of CI on water-saving was significant.

### 3.5. Comprehensive Assessment of GWP, GHGI, and WP

From the above analysis, we know that CI increased N_2_O emissions while reducing CH_4_ emissions compared with FI. For the total GWP caused by CH_4_ and N_2_O under the same N application, the GWP of CI+N3, CI+N2, CI+N1, and CI+N0 decreased by 37.01%, 46.12%, 18.98%, and 27.93% compared with FI+N3, FI+N2, FI+N1, and FI+N0, respectively (Table 2). It can be seen that GWP produced by CI was significantly less than that by FI. By comparing the proportion of GWP produced by CH_4_ and N_2_O, the greenhouse effect of CH_4_ accounted for more than 96% of total GWP. Therefore, CH_4_ was still the main greenhouse gas produced in paddy fields in the cold region. By comparing the relationship between GWP and N application in the two irrigation modes, it was found that the trend of GWP with the increase of N application was basically the same as the trend of CH_4_ with the increase of N application. This may also be because GWP produced by CH_4_ accounted for a large proportion of total GWP. By comparing with the amount of total GWP in the two irrigation modes, we found that the minimum GWP between CI treatments was CI+N0 (3357.09 kg·hm^−2^), at 17.49%, 11.22%, and 14.64% lower than that of CIN1, CIN2, and CIN3, respectively. Minimum GWP among FI was FIN0 (4658.01 kg·hm^−2^), at 7.25%, 33.63%, and 25.40% lower than that of FIN1, FIN2, and FIN3, respectively. Treatments with no N application had the smallest GWP under two irrigation modes; however, in the actual production process of rice, N fertilizer need to be applied to ensure yield. Therefore, GHGI is introduced to balance the contradiction between reducing GWP and ensuring yield affected by applying N fertilizer.

GHGI is an important indicator for evaluating greenhouse-gas production per unit rice yield. According to Table 2, the GHGI of CI+N3, CI+N2, CI+N1, and CI+N0 decreased by 49.86%, 61.23%, 33.53%, and 31.71%, respectively, compared with FI+N3, FI+N2, FI+N1, and FI+N0 under the same N application. This indicated that the greenhouse gas produced by CI per unit yield was significantly less than that of FI. Among CI treatments, GHGI generally decreased with increasing N application rate. CI+N2 had the minimum GHGI (0.37 kg CO_2_-eq·kg^−1^), at 4.90%, 33.69%, and 32.47% lower than CI+N3, CI+N1, and CI+N0, respectively. Among FI treatments, GHGI first increased and then decreased with the increase of N application. FIN3 had the minimum GHGI (0.78 kg CO_2_-eq·kg^−1^), which was 18.69%, 7.56%, and 3.27% lower than FI+N2, FI+N1, and FI+N0, respectively, but it was still 40.58% larger than CI+N2. By comparing the yield and GHGI of each treatment, we can see that CI+N2 treatment has the best emission-reduction effect under the premise of ensuring yield.

According to Table 3, the WP of each treatment was compared. Under the same N application, the WP of CI+N3, CI+N2, CI+N1, and CI+N0 was 77.26%, 92.47%, 116.85%, and 92.96% higher than that of FI+N3, FI+N2, FI+N1, and FI+N0, respectively. It can be seen that CI can significantly increase the WP of rice compared with FI. By analyzing the effect of N application on WP under the two irrigation modes, we found that the WP of each treatment under CI first increased and then decreased with the increase of N application. CI+N2 had the maximum WP (2.05 kg·m^−3^), at 5.12%, 35.14%, and 40.96% higher than that of CI+N3, CI+N1, and CI+N0, respectively. Under FI, the WP of each treatment increased with the increase of N application; FI+N3 had the maximum WP (1.01 kg·hm^−2^), at 6.91%, 28.22%, and 23.18% higher than that of FI+N2, FI+N1, and FI+N0, respectively. By comparing the yield and WP of each treatment, we can see that the yield-increasing and water-saving effect of CI+N2 treatment was significantly higher than other treatments. After comprehensively analyzing the yield, GHGI, and WP of each treatment, it was found that the CI+N2 treatment has the best effect on increasing yield, reducing emissions, and saving water. Its yield, GHGI, and water productivity were 10,224.4 kg·hm^−2^, 0.37 kg CO_2_-eq·kg^−1^, and 2.05 kg·m^−3^, respectively.

## 4. Discussion

### 4.1. Effects of Different Water and N Management Types on CH_4_ and N_2_O

Among many agricultural practices, water management has been recognized as one of the most promising approaches to reduce CH_4_ emissions [23]. CI exerted a significant impact on CH_4_ emission. No water layer in CI was established on paddy fields at each growth stage except for the regreening stage. Soil aeration was greatly increased compared with FI. Therefore, it increased the oxidation rate of CH_4_ and inhibited the activity of methanogen, effectively reducing CH_4_ emissions [24]. This result is in agreement with previous studies that showed that the frequency of soil wetting and drying determined CH_4_ mitigation potential [25,26]. In the present study, later tillering drainage was responsible for the relatively lower CH_4_ emission flux in the stage after tillering for both CI and FI. This is similar to the results of Tariq et al., which could be attributed to the subsequent reduced methanogenesis activity [26]. The present study indicates that the increase of soil-water content promotes CH_4_ emission flux under CI, which agrees with the previous study in that CH_4_ fluxes exhibit an increased trend with the increase of water filled pore space, and excessively low water content promotes soil oxidation [27]. N_2_O is the product of soil nitrification and denitrification. Frequent flooding and drainage of soil may stimulate N_2_O emissions by nitrification and denitrification [7,28]. A large number of studies have shown that there was a trade-off relationship between the emission of N_2_O and CH_4_ in paddy fields [18,29]. In this experiment, the CH_4_ flux sharply decreased during the drying and wetting alternation period, while an N_2_O flux peak occurred, especially in the late tillering drainage period; this was the same as the existing emission pattern [18]. Moreover, the improved diffusion of N_2_O can also be promoted by soil aeration [30]. Studies have shown that CI can significantly reduce GWP produced by N_2_O and CH_4_ by 59.1% compared with FI [24]. In this paper, CI decreased GWP by 46.12% compared with FI under the conventional N application rate (110 kg·hm^−2^). This may be caused by the difference between climate, soil, and water management in different experiment areas.

At present, the results of research on the effect of N fertilizers on CH_4_ emissions in paddy fields are very inconsistent [31]. It was reported that N fertilizers can promote or inhibit the emission of CH_4_ [11,12]. In this study, under CI, N application could significantly increase CH_4_ emissions compared with no N treatment, but there was no significant difference between different N application treatments. Under FI, the treatment of 85 and 110 kg·hm^−2^ N application significantly increased the emission of CH_4_ compared with the treatment with no N application, but there was no significant difference between the application of 110 and 135 kg·hm^−2^ N. This may be because urea promoted the root development of rice, and provided a precursor matrix for the production of CH_4_ in paddy fields. The competitive effect of NH_4_^+^ hydrolyzed by urea on CH_4_ promoted CH_4_ emissions. However, N also promoted the activity of CH_4_-oxidizing bacteria, thus reducing CH_4_ emissions [32]. The difference between N treatments was only 25 kg·hm^−2^ in this experiment, which was a small interval. Therefore, the difference of the N fertilizer may have no significant effect on the inhibition or promotion of CH_4_ emissions. The N fertilizer not only affected soil nitrification and denitrification, but also affected the growth of rice plants and the transport of N_2_O from the soil to the atmosphere. Finally, it had a promoting effect on N_2_O emissions [33,34]. Shcherbak et al. [35] performed a meta-analysis of 78 articles with at least three N application levels in the world, and found that the amount of N_2_O emission increased exponentially with the increase of N application. By fitting the N amount and N_2_O emissions in this experiment, we found that the exponential fitting effect is better than that of the linear one, which was the same as previous research results.

This study was carried out in high latitudes and cold regions of China. Results were quite different from those in other regions because of the different climate, soil conditions, and tillage systems. The results of this study showed that CH_4_ and N_2_O emissions were 329.48 and 0.32 kg·hm^−2^ under FI, and 174.83 and 0.35 kg·hm^−2^ under CI. A study by Li et al. showed that CH_4_ emissions from early rice and late rice were 190.7 and 238.4 kg·hm^−2^, and N_2_O emissions were 1.3 and 1.7 kg·hm^−2^ under FI, respectively, in Hubei Province (30°21′ N; 112°09′ W), China [10]. CH_4_ emissions of the present study were higher than the season emissions of early rice or late rice, but lower than their annual CH_4_ emissions. N_2_O emissions of the present study were much lower than the results of Li et al. They applied 165 and 180 kg N·hm^−2^ for early rice and late rice, respectively, to ensure the yield due to their lower total N in the soil, while our moderate N application rate was 110 kg·hm^−2^. More N fertilizer promoted N_2_O emissions in southern China. On the whole, CH_4_ and N_2_O emissions from paddy fields in cold regions were lower than those in southern China. Pandey et al. reported that CH_4_ and N_2_O emissions in Vietnam were 108 and 0.31 kg·hm^−2^, respectively, under FI, with 100 kg N·hm^−2^ [8]. CH_4_ emissions were lower than those in the present study due to the relatively shorter growth period of rice (84 days) in their study. A two-year experimental study of Yang et al. in China’s Jiangsu Province (34°63′21” N, 121°05′22” E) showed that the average CH_4_ emissions under CI and FI were 114.5 and 425 kg·hm^−2^, respectively, and average N_2_O emissions were 4.84 and 1.99 kg·hm^−2^, respectively. Compared with FI, CI reduced CH_4_ emissions by 73%, but increased N_2_O emissions by 125% in southern China [36], while in this experiment, CI reduced CH_4_ emissions by 47%, but increased N_2_O emissions by only 9%. CI had a better CH_4_ emission-reduction effect in southern China than in this experiment. However, the increase of N_2_O under CI was larger than that in the present experiment. This may be due to different soil basic properties and nitrogen-application rate. In the experiment of Yang et al., the soil organic matter and total nitrogen values were only 52% and 7% of those this study, respectively, and they applied more N fertilizer than in this study. Lower soil organic matter and higher N fertilizer application under CI inhibited CH_4_ emissions, but promoted N_2_O in southern China. Although the effect of CH_4_ emission reduction under CI in the paddy fields was not as good as that in the southern China, the increase of N_2_O was much smaller.

### 4.2. Effects of Different Water and N Management Types on Yield, WP, and GWP

FI is the most common irrigation mode for farmers. However, in this study, compared with FI, we saw that CI can effectively increase yield by 5.53~38.97%. This may be because CI could effectively control ineffective tillering, reduce N loss caused by ineffective tillering, and increase grain number per spike, seed-setting rate, 1000-grain weight (Table 3), and the development of rice-root systems [37]. FI needs to keep a thick water layer in the field, which increases the waste of water resources caused by evaporation, surface runoff, and deep seepage. The water-saving effect of CI was significant from the tillering stage until the maturity stage (Figure 4). The management mode of a no-water layer in the field reduced the loss of water, N fertilizer, and unnecessary water pollution produced by the paddy fields [38]. The water-saving and yield-increasing characteristics of CI led to an overall increase of WP. At the same time, CI reduced the amount of irrigation water, which can effectively reduce the labor cost and fuel consumption of farmers. Promoting the popularization of CI could effectively solve the problem of water shortage in rice-growing areas in cold regions. In this experiment, the results of the comprehensive evaluation of GWP, GHGI, and WP in CIN3 and CIN2 were all good, but the higher N application rate would reduce N use efficiency, resulting in unnecessary N loss and an increase of N_2_O emissions [39,40].

CI could effectively reduce the GWP produced by N_2_O and CH_4_ in paddy fields. Results showed that CI+N2 was better than other treatments on reducing greenhouse-gas emissions. According to Heilongjiang Statistical Yearbook 2016 [8], the rice-planting area in Heilongjiang is about 3.81 × 10^6^ hm^2^. If the irrigation and N application mode of CI+N2 recommended in this experiment was used to replace the conventional FI+N2 treatment, it is estimated that the annual GWP could be reduced by about 1.24 × 10^10^ kg CO_2_-eq, and irrigation water would be reduced by 1.07 × 10^10^ m^3^ during the growth period of rice. The application of CI in rice-planting areas in cold regions is of great significance for reducing greenhouse-gas emissions, alleviating the shortage of water resources and ensuring high yield of rice.

## 5. Conclusions

Under the two irrigation modes, N application could significantly increase CH_4_ emissions in paddy fields compared with no N application treatments. However, there was no significant difference between 85, 110, and 135 kg·hm^−2^ N application treatments under CI, and 110 and 135 kg·hm^−2^ N application treatments under FI; N_2_O emissions in paddy fields significantly increased with the increase of N application rate under both CI and FI. Compared with FI under the same N application, CI significantly reduced CH_4_ emissions by 19.42~46.94%, but at the same time increased N_2_O emissions by 5.66~11.85%, finally leading to a result of the total GWP of CI treatments being significantly less than that of FI treatments. Appropriate N application could increase yield components. Under the same N application, the spikes per unit area of CI rice were significantly smaller than those of FI, but the grain number per spikes, seed-setting rates, and 1000-grain weight were larger than those of FI, which made up for the loss of smaller spikes per unit area and increased yield by 5.53~38.97%. Compared with FI, CI significantly reduced water consumption at each growth stage (except for the regreening stage) and total water consumption. The GHGI of different CI treatments was significantly lower than that of FI treatments, and the WP of CI treatments was significantly higher than that of FI treatments. Through comprehensive comparison and analysis, CI+N2 treatment (CI with 110 kg·hm^−2^ N application) is recommended for rice-planting areas in cold regions because of its highest yield and WP, and lowest GHGI, meeting the purposes of water-savings, emission reductions, and high rice yield.

## Figures and Tables

**Table 1:** Water management of different irrigation modes.

Irrigation Modes	Regreening (mm)	Former Tillering	Middle Tillering	Later Tillering	Jointing and Booting	Heading and Flowering	Milky Maturity	Yellow Maturity
CI	0 ~30	0.7 θs ~0 mm	0.7 θs ~0 mm	drainage	0.8 θs ~0 mm	0.8 θs ~0 mm	0.7 θs ~0 mm	drying
FI	0 ~30	0 mm ~50 mm	0 mm ~50 mm	drainage	0 mm ~50 mm	0 mm ~50 mm	0 mm ~50 mm	drying

Note: Data before “~” are the lower limits of water, data after “~” are the upper limits of water. CI, controlled irrigation. FI, flood irrigation, θs, saturated moisture content of root layer soil, same as below.

**Table 2:** Greenhouse-gas intensity and water productivity of each treatment.

Treatments	CH_4_ Emission (kg·hm^−2^)	N_2_O Emission (kg·hm^−2^)	GWP by CH_4_ (kg CO_2_-eq·hm^−2^)	GWP by N_2_O (kg CO_2_-eq·hm^−2^)	Total GWP (kg CO_2_-eq·hm^−2^)	GHGI (kg CO_2_-eq·kg^−1^)	Water Consumption (kg·hm^−2^)	WP (kg·m^−3^)
CI+N0	157.77e	0.14e	3313.19e	43.90e	3357.09f	0.55d	4224g	1.45c
CI+N1	189.79d	0.27d	3985.53d	83.07d	4068.60e	0.56d	4819f	1.51c
CI+N2	174.83d	0.35bc	3671.44d	109.90bc	3781.34e	0.37f	4998e	2.05a
CI+N3	180.84d	0.44a	3797.65d	135.08a	3932.73e	0.39e	5197d	1.95b
FI+N0	219.83c	0.13e	4616.46c	41.55e	4658.01d	0.80bc	7095c	0.82f
FI+N1	235.54b	0.24d	4946.30b	75.60d	5021.90c	0.84b	7610b	0.79f
FI+N2	329.48a	0.32c	6919.04a	99.41c	7018.45a	0.95a	7799ab	0.94e
FI+N3	291.56a	0.39b	6122.83a	120.77b	6243.60b	0.78c	7982a	1.01d

Note: Lowercase letters represent the differences between each treatment (*p* < 0.05), N0, N1, N2 and N3 represent 0 kg·hm^−2^, 85 kg·hm^−2^, 110 kg·hm^−2^ and 135 kg·hm^−2^,respectively, same as below. GWP: global warming potential; GHGI: gas emission intensity; WP: water productivity.

**Table 3:** Yield and its components in each treatment.

Treatments	Spikes Per Unit Area	Grain Number Per Spike	Seed-Setting Rate/%	1000-Grain Weight/g	Yield (kg·hm^−2^)
CI+N0	406e	74c	84.71c	26.58c	6130.12d
CI+N1	510d	84bc	88.99b	27.33bc	7294.96c
CI+N2	554c	100a	91.82a	28.32a	10,224.4a
CI+N3	541c	89b	92.92a	27.01bc	10,113.2a
FI+N0	472de	70c	83.20c	26.09c	5808.84d
FI+N1	547c	73c	86.92bc	26.66c	5985.3d
FI+N2	588b	90b	90.40ab	27.65b	7357.2c
FI+N3	608a	89b	88.21b	27.72b	8049.78b

Note: Lowercase letters represent the differences between each treatment (*p* < 0.05).
